# A CRISIS LEADERSHIP FOR NURSE MANAGERS IN CRISIS SITUATIONS: DEVELOPMENT AND PSYCHOMETRIC EVALUATION

**DOI:** 10.1093/geroni/igae098.2641

**Published:** 2024-12-31

**Authors:** Sun Ju Kim

**Affiliations:** Chungnam National University Se-Jong Hospital, Se-Jong, Republic of Korea

## Abstract

Crisis situations such as COVID-19 have a negative impact on society and the economy, with increased mental health issues, medical crises, and mortality among nurses. The inadequate leadership of nurse managers during crisis situations can negatively impact the quality of patient care and infection transmission. Consequently, the aims of this study were to develop a crisis leadership scale for nurse managers and to evaluate its psychometric properties. For the development of the initial items, the literature was reviewed along with the existing measuring tools. This study was conducted from August to October 2023. A total of 443 nurse managers in 34 hospitals in Korea participated in the study to verify the validity and reliability of the instrument. The exploratory and confirmatory factor analyses yielded a five-factor solution, while the confirmatory factor analysis showed a good fit (RMSEA =.07, SRMR =.02, CFI =.92, TLI =.91). Therefore, the final instrument was derived, containing 28 items on “crisis management competency” (12), “decision-making” (6), “collaboration” (4), “communication” (3), and “building trust” (3), as well as 5 factors, scored on a 5-point Likert scale. The Cronbach’s α for this instrument was.96. In conclusion, the crisis leadership scale showed good psychometric properties for four validity metrics (content, construct, convergent, discriminant and criterion validity) and internal reliability. These findings indicate that the instrument can be applied in both research and clinical practice, being capable of quantitatively measuring the degree of crisis leadership skills of nurse managers.

